# Mental health in diabetes care. Time to step up

**DOI:** 10.3389/fcdhc.2022.1039192

**Published:** 2022-10-13

**Authors:** Frank J. Snoek

**Affiliations:** Department of Medical Psychology, Amsterdam Public Health Research Institute, Amsterdam University Medical Centers, Vrije Universiteitr, Amsterdam, Netherlands

**Keywords:** mental care, diabetes care, screening, well-being, depression

## Introduction

It is well-recognized that diabetes is a psychologically burdensome chronic condition, not in the least due to the continuous need for behavioral self-regulation. Diabetes self-management is relentless and extends beyond striving to keep glucose levels within range. As noted by the sociologists Corbin and Strauss, living with a chronic condition involves ‘three lines of work’: illness work, everyday life work, and biographical work (1). Indeed, the person diagnosed with diabetes faces multiple adaptive tasks beyond glucose control, such as dealing with the medical system, managing stress, keeping emotional balance, adjusting to physical limitations, and maintaining role functioning (2). Much of that work is largely invisible to others, including professionals whose attention often is on ‘the numbers’ rather than the person. Whereas the majority of people affected by diabetes learn to adapt over time and lead a productive life, roughly one-third of people living with diabetes at some point experience clinically relevant symptoms of emotional distress, ranging from ‘normal’ adjustment problems to more pervasive psychological problems, including anxiety, depression and eating disorders (3, 4). Epidemiological research has convincingly shown that people with type 1 and type 2 diabetes have a two-fold increased risk of developing clinical depression relative to the general population, where the point prevalence is between 5 and 7% (5, 6). As to the etiology of depression in diabetes there are still many unknowns, but a higher prevalence of depression has also been found in other medical chronical conditions, speaking to the ‘hardship’ hypothesis (7). This theory postulates that poor mental health in people with a chronic medical illness results primarily from the psychological and economic burden of living with a disease. Alternatively, shared underlying biological factors (e.g. inflammation) may be implicated (8). Irrespective of etiology, common mental health disorders such as anxiety and depression, can be effectively treated, although effects on glycemic outcomes are modest (9).

## Guidelines

There is consensus that health care professionals have a responsibility when it comes to monitoring and addressing the emotional needs of people with diabetes (10). A substantial proportion of serious psychological distress, however, remains unrecognized and untreated (11). This finding should not surprise us, as diabetes doctors in busy clinics have very limited time. While most patients do wish to discuss their emotional health with their diabetes care provider, some patients may be reluctant to disclose mental problems and prefer to discuss these with family and friends or a professional outside the diabetes clinic (12),. To improve the recognition and management of mental health in diabetes, it needs to be made integral part of the ‘system’. Guidelines recommend periodic psychological screening and monitoring as part of clinical routine, using standardized questionnaires (13).. The ADA Position Statement on Psychosocial Care recommends screening at the initial visit, at periodic intervals (e.g. 3-monthly consultations) and when there is a change in disease, treatment, or life circumstance (14). It is recommended to include caregivers and family members in the assessment. Preferably a clinical psychologist is member of the team, to advise and supervise the team, and offer psychological treatment to those in need. We lack exact data, but it would appear that these recommendations are often not met in clinical practice, for a number of reasons, mainly lack of time and resources (15). Moreover, not all professionals feel comfortable discussing mental health issues and are unsure what to do when patients report serious emotional distress. Implementing a psychological screening procedure in busy clinics may come with logistical problems, and can be perceived as disrupting normal clinical routine. Moreover, time invested in mental health screening is often not reimbursed as part of standard care. Also, few diabetes care teams have a clinical psychologist in the care team, and referring patients to a mental health clinic can be complicated, as many mental health clinics have waiting lists and often lack diabetes expertise. This can make mental health workers hesitant to accept clients with ‘diabetes problems’ and refer them back. These barriers are not easily overcome and require organizational enhancements and sufficient funding. Here I discuss the need for transform clinical practice based on collaborative care, where diabetes and mental health experts work closely together and mental health is integral part of ongoing diabetes care. In this context I suggest shifting the focus of screening to case-finding based on risk stratification. The vast majority of patients do not suffer from a mental illness, but rather experience ‘normal’ coping difficulties for which monitoring of wellbeing and “light” psychological interventions will suffice. In the face of the ever-growing population of people with diabetes and limited resources, evidence-based e-mental health interventions should be adopted to increase access to psychological care at relatively low costs.

## Problems and disorders

Based on symptomatology and level of impairment, we can conclude that most mental health problems of people with diabetes can be classified as mild or subclinical, not meeting DSM or ICD diagnostic criteria for a psychiatric condition. Yet, mild problems are clinically relevant, as they can stand in the way of healthy coping with diabetes and can progress to more serious disorders. However, they do not require psychiatric treatment. Coping or adjustment problems, often referred to as diabetes-related distress or simply diabetes distress, affect roughly one-third of people with type 1 and type 2 diabetes (4, 16). Diabetes distress can be experienced by patients at any time, but most likely to develop after diagnosis, following a critical incident, e.g. DKA or severe hypoglycemic event, and when confronted with diabetes complications (17). It is important to acknowledge that diabetes distress is not pathological, but rather a psychological response to the strains of living with diabetes and not per se maladaptive. Prolonged high diabetes distress however constitutes a risk factor for demoralization or ‘diabetes burnout’, decreased patient engagement and consecutive suboptimal glycemic outcomes (18). Research shows that evidence-based self-management support programs, psychoeducation and peer support can effectively help to alleviate distress (19). Diabetes distress is not captured by depression screeners such as the CES-D or PHQ-9, but can be reliably assessed using validated questionnaires, such as the Problem Areas In Diabetes (PAID) or Diabetes Distress Scale (DDS) (19). These self-report measures help to identify individual’s diabetes-specific sources of distress and provide norm data and a cut-off to identify low, moderate, and high distress (20). More longitudinal research is needed regarding different trajectories of diabetes distress, but data suggest that - without extra support- high distress remains stable over time in approximately one-third of those with high baseline distress (21, 22). Periodic monitoring of diabetes distress is therefore advised and fits well in routine diabetes consultations (23, 24). In sum, it is important to distinguish problems (diabetes distress) and disorders (mental illness). And while both deserve to be addressed as part of ongoing diabetes care, they require a differential approach, where much of the support can be delivered by well-trained diabetes professionals, both individually and in groups. Clinical psychologists with diabetes expertise can provide training and supervision to care teams. Psychological disorders require a referral to specialist mental health care, either within or outside the diabetes clinic.

## Screening

The recommendation to screen all patients with diabetes for mental health problems periodically, builds on the notion that 1) as a group, people with diabetes are at increased risk for mental health problems such as depression, anxiety and eating disorders, and 2) that otherwise mental problems would stay largely undetected, and 3) timely identification translates in offering effective treatment for those in need, and thereby improve prognosis and consequently better health outcomes. The focus in screening most often is on depression. However, there is reason to question the utility of routine depression screening in all people with diabetes, as the vast majority of people with diabetes have no depression. Universal screening for depression in medical care is unlikely to be cost-effective when not coupled with organizational enhancements (25). These enhancements would include, at minimum, securing a follow-up for all positive screens and having effective referral and care pathways in place. As we demonstrated, asking persons with diabetes to fill out a depression screener with written feedback in itself, does not improve outcomes relative to care as usual (26). It makes sense to rethink the screening paradigm and shift to case-finding as a more economic and appropriate approach in diabetes clinics. Case-finding aims to pre-identify persons at high risk for mental illness within a certain population, based on known risk factors (27). Research has identified several risk factors for the onset and chronicity of depression in the general population, including female gender, younger age, previous depressive episodes, major life events, a family history of depression, and social deprivation (28). Specific to diabetes, depression is known to be increased in persons with prolonged elevated HbA1c levels (despite intensive treatment and support) and manifest diabetes complications (29). Reviewing known risk factors as part of the initial clinical evaluation, can help to pre-identify patients at risk, and tailor follow-ups and care pathways accordingly. We can estimate up to 10% to 20% of all patients to be classified as at-risk and – if wished for and untreated – should be referred to a mental health specialist. The risk-profile is also important in matching diabetes treatment and education to the person’s specific capacities and needs to achieve best possible outcomes. For example, taking into account comorbid depression or an eating disorder can impact the choice of medication, insulin regimen and diabetes technology. With a psychologist on the team, the psychological and self-management implications of the mental health problems can be reviewed and help inform shared decision-making. The vast majority of patients will not show to be at-risk for mental illness, and for them periodic monitoring of wellbeing will suffice. Of course, persons initially assessed as being at low risk, can develop a (new) mental disorder in due course, which may or may not be related to the diabetes. It is thus important to be alert to critical moments and transitions, e.g. the onset of complications and major life events, that may increase a person’s psychological vulnerability.

## Well-being

As noted, most people with diabetes do not suffer from a mental illness. However, it is common for people with diabetes to experience some level of distress, with concerns related to living with diabetes and its ramifications. We should be mindful of the fact that negative emotions are not per se in need of a ‘fix’. In most cases people learn to self-manage their emotions, with help of family, friends and supportive health care providers. In this context, enhancing self-awareness, understanding, and normalizing emotional reactions are helpful strategies to help restore emotional equilibrium. In line with the World Health Organization’s definition of health, emotional wellbeing is more than the absence of mental illness (30). Screening out depression or anxiety thus is not sufficient nor appropriate to evaluate a person’s wellbeing. Monitoring of wellbeing in diabetes is focused on reviewing the persons’ perceived quality of life across different life domains and elicit specific needs related to coping with diabetes. The role of the health care provider lies primarily in active listening, showing empathy and helping patients identify strategies how to best self-manage the diabetes while living a full life (31). Validating a person’s emotions in itself can make the patient feel understood and help alleviate feelings of diabetes distress (32). This does not necessarily require administering a validated questionnaire, but using short, patient-friendly measures such as the WHO-5 Wellbeing Index (WHO-5) can facilitate the wellbeing conversation and track changes over time. A specific advantage of using the WHO-5, is that it is positively worded, yet flags likely depression when the score is below a cut-off, warranting diagnostic follow-up (33). Finally, in the context of value-based care, standardized assessment of patient-reported outcomes (PROs) contributes to quality improvement (34).

## Future directions

Improving mental health in diabetes care requires a collaborative care model, with diabetes and mental health expertise effectively integrated, offering patients the best of both worlds (35). It requires a psychologist to be member of the team, or at least to be available for referral and consultation. To address the shortage of diabetes psychologists, training programs for psychologists are called for, as have been successfully developed in collaboration with national psychology and diabetes associations, for example in the USA (36).

We can expect an integrated approach to diabetes care to help improve both medical and patient-reported outcomes, and consequently cost-saving (35). More implementation research is warranted to test innovative approaches to case-finding, using Artificial Intelligence (AI) and adaptive computerized testing to help differentiate persons’ risk for mental illness and offer tailored interventions (36). This fits well with advancing personalized medicine, aimed to tailor timing of monitoring and treatments to the needs of patients as they evolve over time. Recent advancements in ‘digital phenotyping’ by means of ecological momentary assessments (EMA), using smartphone technology, have shown promising results (37). Evidence-based psychological interventions have been developed for a range of psychological problems, including internet and mobile-based programs (IMI) (38). Integrating IMI’s in diabetes care can help to provide psychological support to large groups of patients. Self-guided applications may assist patients in improving healthy coping and prevent symptoms of emotional distress from worsening (39). Mental vitality is a conditio sine qua non for successful diabetes self-management. There is no health without mental health (40).

## Author contributions

FS wrote the MS. The author confirms being the sole contributor of this work and has approved it for publication.

## Conflict of interest

The author declares that the research was conducted in the absence of any commercial or financial relationships that could be construed as a potential conflict of interest.

## Publisher’s note

All claims expressed in this article are solely those of the authors and do not necessarily represent those of their affiliated organizations, or those of the publisher, the editors and the reviewers. Any product that may be evaluated in this article, or claim that may be made by its manufacturer, is not guaranteed or endorsed by the publisher.
